# Clinical Outcomes of Endoscopic Submucosal Dissection for Early Esophageal Squamous Cell Neoplasms: A Retrospective Single-Center Study in China

**DOI:** 10.1155/2016/3741456

**Published:** 2016-08-07

**Authors:** Yanfang Chen, Ye Zhao, Xiaojing Zhao, Ruihua Shi

**Affiliations:** ^1^Department of Gastroenterology, Zhongda Hospital, Southeast University, No. 87 Dingjiaqiao Road, Nanjing, Jiangsu 210009, China; ^2^Department of Gastroenterology, The First Affiliated Hospital of Nanjing Medical University, No. 300 Guangzhou Road, Nanjing, Jiangsu 210029, China

## Abstract

*Aims*. To retrospectively analyze the clinical outcomes for a large number of endoscopic submucosal dissections (ESDs) in early esophageal squamous cell neoplasms (ESCNs) at the First Affiliated Hospital of Nanjing Medical University.* Patients and Methods*. From January 2010 to February 2014, 296 patients (mean age 61.4 years, range 31–85 years; 202 men) with 307 early ESCNs (79 intramucosal invasive esophageal squamous cell carcinomas (ESCCs) and 228 high-grade intraepithelial neoplasia (HGIN) cases) were included from a total of 519 consecutive patients who were treated by esophageal ESD at our hospital. The primary end points of the study were rates of en bloc resection and complete resection. Secondary end points were complications, residual and recurrence rates, and mortality during follow-up.* Results*. The en bloc resection rate and complete resection rate were 93.5% and 78.2%, respectively. Complications included strictures (8.4%), perforations (1.0%), and bleedings (0.7%). Twenty-seven (9.1%) patients experienced residual and 18 (6.1%) patients experienced recurrence during a mean follow-up period of 30 months. Thirteen patients died from causes unrelated to ESCC, and no cancer-related death was observed.* Conclusions*. Our study showed that ESD is a successful and relatively safe treatment for intramucosal invasive ESCC and HGIN, fulfilling the criteria of lymph node negative tumors. This should encourage clinicians to select ESD performed by experienced operators as a potential or even preferred treatment option for lesions amenable to endoscopic treatment.

## 1. Introduction

Early-stage esophageal squamous cell neoplasms (ESCNs) are increasingly recognized and detected by endoscopists, due to improvements in endoscopic image quality and increased education and awareness of these often flat lesions [1–3]. The link between intraepithelial neoplasia and invasive cancer is well established and esophageal squamous cell carcinoma (ESCC) is currently the dominant histological type of all esophageal carcinomas in some Asian countries, including China [4]. In early esophageal neoplasia, endoscopic resection has been increasingly conducted around the world, which aims to maintain the integrity of the esophagus and avoid the high morbidity and mortality rates of surgical treatment [5], and has been shown to be effective. Endoscopic mucosal resection (EMR) was the first developed endoscopic resection strategy. However, en bloc resection with EMR is limited to small lesions, and piecemeal resections are frequent. Histological evaluation is inconclusive or incorrect after piecemeal resection, and piecemeal resection is known to be a risk factor for recurrence [6, 7]. Endoscopic submucosal dissection (ESD) is a new resection technique that allows en bloc resection of gastrointestinal lesions regardless of their location and size. En bloc resection improves the histological assessment of the specimens and can reduce the risk of recurrence. ESD was initially developed in Japan, to improve endoscopic treatment of early gastric cancer [8, 9], and has been shown to be superior to conventional EMR in terms of en bloc resection rate and recurrence rate [10]. However, some studies showed that ESD could have a higher rate of complications, such as bleeding, perforation, and postoperative esophageal stricture resulting from complicated procedures [11]. Although ESD has grown popular in resecting neoplasms in the stomach, the application of ESD to the esophagus has been limited by greater technical difficulty. Many articles have reported the treatment results of ESD, but most of the publications only include small numbers of cases and have short follow-up periods. Since most of the studies were conducted in Japan, it can hardly represent the basic characteristics of patients worldwide. The aim of this retrospective study was to analyze the clinical outcomes for a large number of ESDs in early ESCNs at our hospital.

## 2. Patients and Methods

**Figure 1:**
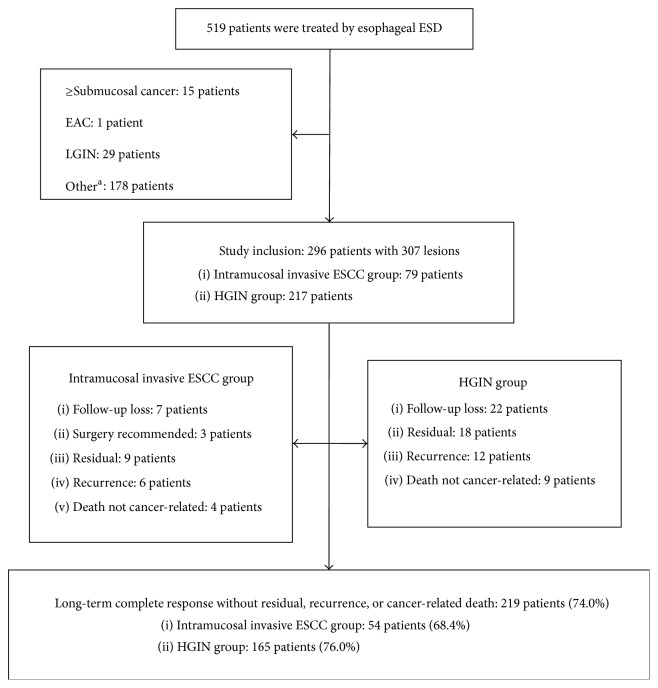
Flow diagram for the study patients. ESD: endoscopic submucosal dissection; EAC: esophageal adenocarcinoma; LGIN: low-grade intraepithelial neoplasia; ESCC: esophageal squamous cell carcinoma; and HGIN: high-grade intraepithelial neoplasia. ^a^Main including leiomyoma, stromal tumor, inflammation, and epithelial hyperplasia.

From January 2010 to February 2014, 519 consecutive patients who provided written informed consent were treated by esophageal ESD at the First Affiliated Hospital of Nanjing Medical University. Among them, 296 patients who met the following criterion were included in the present retrospective study: a histologically confirmed diagnosis of intramucosal invasive ESCC or high-grade intraepithelial neoplasia (HGIN) on the ESD specimen (Figure 1). This study was approved by our institutional review board (IRB number: 2014-SR-198).

According to a center protocol, before ESD was performed all patients at high risk of esophageal neoplasia underwent (1) CT of the chest; (2) high-resolution video endoscopy and chromoendoscopy with Lugol staining, and occasionally magnifying endoscopy with narrow-band imaging; (3) endoscopic ultrasonography; and (4) endoscopic biopsy. Patients in whom the neoplastic lesion appeared to be confined to the esophageal mucosa with no apparent lymph node metastases were selected for ESD treatment. In cases with submucosal cancer, the incidence of lymph node metastasis is 15.4–44.1% [12]. Current data support surgical resection in the setting of submucosal infiltration by ESCC, unless comorbidity or advanced patient age precluded it.

### 2.1. ESD Procedure

ESD was performed with the patient under general anesthesia. Procedures were performed using a single-channel upper gastrointestinal endoscope with a water-jet system (GIF-Q260J; Olympus). Carbon dioxide was used for insufflation. There were five principal expert endoscopists who had sufficient skill to perform ESD. The ESD procedure was carried out in a standardized way as follows:

1. White light endoscopy and Lugol chromoendoscopy were conducted to improve the visualization of the lesion and determine the resection margin.
2. The resection border was marked about 5 mm from the lesion margin using argon plasma coagulation (APC).
3. The submucosa was injected with a saline solution (0.9% saline with a small amount of indigo carmine and epinephrine). Injections were repeated as needed.
4. A circumferential incision of the mucosa was made outside the marking dots by using a hook-knife, followed by submucosal dissection using an insulated-tip knife. To control bleeding, coagulation of visible vessels or bleeding sites was performed using hemostatic forceps and APC, during ESD and at the end of the procedure. If the muscularis was exposed during ESD, or perforation was suspected, a hemoclip was applied.

After the procedure, intravenous proton pump inhibitor (PPI) (esomeprazole 40 mg every 12 hours or equivalent) for 3 to 5 days, followed by oral PPI (esomeprazole 20 mg twice a day or equivalent) for 4 weeks, was administered to relieve pain, prevent procedure-related bleeding, and promote ulcer healing. Patients without serious symptoms or complications were put on a liquid diet on the 2nd day after ESD, followed by a soft diet the following day, and were then generally discharged within few days. If complications occurred, the schedules were changed according to the individual patient's condition.

### 2.2. Histological Evaluation and Additional Treatment

Resection specimens were stretched and fixed onto a Styrofoam plate with needles. Diameter of specimen was measured and specimens were sent for histopathological assessment. Histological evaluation of the resected specimen was performed according to the World Health Organization classification [13]. Histopathological work-up provided information about histological type, invasion depth, resection margins, and presence or absence of lymphovascular invasion.

Patients with submucosal involvement, lymphovascular invasion, or cancer-positive vertical margins on histological evaluation were recommended to have additional treatment, such as esophagectomy with lymph node dissection or chemoradiation, depending on the risk of metastasis and the patient's condition.

### 2.3. Follow-Up

Follow-up endoscopy was usually performed after 2, 6, 12, and 18 months, and annually thereafter. Lugol spraying was applied during follow-up and biopsy was taken from any area where residual or recurrence was suspected. When biopsies confirmed neoplastic lesions, APC, EMR, ESD, or additional treatment was performed. The starting date of the follow-up was defined as the date of ESD, and the end of the follow-up was either the date of death or the last endoscopic examination.

### 2.4. Definitions

En bloc resection was defined as resection in a single piece as opposed to piecemeal resection (in multiple segments) [14]. Complete resection was defined as en bloc resection with vertical and horizontal margins free of neoplasia at histology.

Complications were defined as bleeding, perforation, or stricture. Bleeding during ESD was considered as a complication when it was severe and resulted in the premature termination of endoscopic resection. Delayed bleeding was defined when clinical bleeding signs were observed after ESD (hematemesis and/or melena or hemoglobin drop >20 g/L) [15]. Perforation was endoscopically diagnosed during the procedure or by the presence of free air on plain chest radiography after ESD [14]. Stricture was judged as a complication when it was symptomatic and required endoscopic treatment [16].

If a neoplasia was found at the previous resection site before 6 months, it was defined as residual. After 6 months, it was defined as local recurrence. In addition, if a new lesion was detected in a different area from the initial neoplasia before 12 months, it was defined as synchronous recurrence. After 12 months, it was defined as metachronous recurrence.

Resection time was defined as the interval from the time of marking the lesion to the time of collecting the specimen after resection [17]. Cancer-related death was defined as death caused by metastatic ESCC (diagnosed by CT).

### 2.5. End Points

The primary end points of the study were rates of en bloc resection and complete resection achieved by ESD in early esophageal neoplasia. Secondary end points were complications related to the ESD procedure, residual and recurrence rates of esophageal neoplasia after ESD, and mortality during follow-up.

### 2.6. Data Collection

The data for the patients and lesions were collected from the medical records that included the patient demographics, the endoscopic characteristics, the details of ESD procedure, the histologic findings of the resected specimens, and the follow-up examinations. Incomplete data were investigated from the telephone contact with patients or their families.

### 2.7. Statistical Analysis

Data management and statistical analysis were performed using PASW Statistics (version 18.0; IBM, Chicago, IL). For descriptive statistics, mean (s.d.) was used in case of a normal distribution of variables; median (interquartile range) was used for variables with a skewed distribution. Numeric values were compared using the *t*-test or the rank sum test, depending on the distribution (normal and nonnormal, resp.). For the comparison of categorical data, the chi-squared test was employed. *P* values of <0.05 were considered to be statistically significant.

## 3. Results

### 3.1. Patient and Lesion Characteristics

The characteristic features of the patients and lesions are listed in Table 1. A total of 296 patients (mean age 61.4 years, range 31–85 years; 202 men) with 307 early ESCNs (79 intramucosal invasive ESCCs and 228 HGIN cases) were included in the study. Patient age (*P* = 0.947), sex (*P* = 0.980), and hot-eating habit (*P* = 0.339) distribution were similar in the intramucosal invasive ESCC and HGIN group.

Overall, 0 lesions were located in the cervical esophagus, 22 (7.2%) in the upper thoracic esophagus, 181 (59.0%) in the middle thoracic esophagus, 100 (32.6%) in the lower thoracic esophagus, and 4 (1.3%) in the abdominal esophagus.

The median diameter of the specimen was 3.0 cm (range 0.8–8.0 cm), being 2.0 cm or larger in 289 (94.1%) specimens. The resected specimen diameter for intramucosal invasive ESCCs was significantly larger than it was for HGIN cases (*P* = 0.008).

Two hundred and twenty-three (72.6%) lesions extended less than one-half of the circumference of the esophageal lumen, and there was significant difference between intramucosal invasive ESCCs and HGINs (*P* = 0.006). Fifty-one (16.6%) lesions extended more than three-fourths of the circumference, and there was also significant difference between them (*P* = 0.006).

### 3.2. Endoscopic Treatment/Patients' Clinical Course after ESD

“Endoscopic treatment/follow-up” is listed in Table 2 and Figure 1. Esophagectomy with lymph node dissection was recommended in 5 patients (2 with cancer-positive vertical margins and 3 with lymphovascular invasion), and no residual cancer or lymph node metastasis was detected in the surgical specimens. Follow-up rate was 90.2% (267/296), and follow-up period was a mean of 30 months (range 10–58 months).

The en bloc resection rate was 93.5% (287/307), and it was significantly higher for HGINs than it was for intramucosal invasive ESCCs (*P* = 0.041). The complete resection rate was 78.2% (240/307); no statistical difference was noted in terms of complete resection rate (*P* = 0.235).

The median resection time was 60 minutes (range 20–240 minutes), and the median duration of hospitalization was 6 days; differences in resection time (*P* = 0.487) and length of hospitalization (*P* = 0.351) showed no statistical significance.

Stricture served as the most common complication with incidence of 8.4% (25/296), followed by perforation 1.0% (3/296) and bleeding 0.7% (2/296). Two patients had more than one complication. All of these 25 patients who experienced esophageal stricture underwent ESD for a lesion that extended more than half the luminal circumference. Of the 51 patients in whom the lesion extended more than three-fourths of the luminal circumference, stricture was observed in 23 patients (45.1%). All the postprocedure strictures were successfully managed with balloon dilation (median 4 sessions, range 1–12 sessions) and/or placement of stent. Perforation occurred in 3 patients, and all of the perforations were successfully managed endoscopically. Bleeding during ESD that was severe and resulted in the premature termination of endoscopic resection was not seen. Delayed bleeding occurred in 2 patients (1 and 12 days after ESD, resp.). One patient received successful endoscopic hemostasis, and one patient had a good evolution with conservative treatment. Transfusion of red blood cells was not necessary in any patient.

Residual and recurrence were diagnosed histologically by biopsies. Twenty-seven (9.1%) patients experienced residual and 18 (6.1%) patients experienced recurrence during follow-up. There were no significant differences in residual and recurrence rates between the intramucosal invasive ESCC and HGIN group. Of these 27 patients with residual, 23 patients were treated by APC, 3 patients were treated by second ESD, and 1 patient was treated by esophagectomy. Of these 18 patients with recurrence, 12 patients were treated by APC, 3 patients were treated by second ESD, 2 patients were treated with chemoradiation, and 1 patient was treated by esophagectomy. However, no residual or recurrence was observed thereafter. Thirteen patients died from causes unrelated to ESCC, and no cancer-related death was observed. Long-term complete response without residual, recurrence, or cancer-related death was obtained in 219 patients (74.0%).

## 4. Discussion

To our knowledge, this study represents one of the largest esophageal ESD cohorts yet reported. The en bloc resection rate and complete resection rate were good. Follow-up rate was high, in excess of 90%, and follow-up period was a mean of 30 months. Complication rates were low, ranging from 0.7% to 8.4%. Twenty-seven (9.1%) patients experienced residual and 18 (6.1%) patients experienced recurrence during follow-up. Thirteen patients died from causes unrelated to ESCC, and no cancer-related death was observed. A total of 219 patients (74.0%) obtained a long-term complete response without residual, recurrence, or cancer-related death.

The en bloc resection rate was similar to other series of esophageal ESD, where rates varied between 90 and 100% [14, 16, 18, 19]. However, complete resection rate seemed to be lower than previously reported studies, where rates varied between 80 and 94.6% [14, 17, 20, 21]. There may be at least 1 main explanation for such a rate. The definition of complete resection was very strict in the current study, and resections showing low-grade intraepithelial neoplasia (LGIN) in the margins of the specimen were classified as incomplete resection. In some other studies, complete resection of cancer was defined when the specimen showed margins free of cancer, which means that HGIN/LGIN-containing margins were not considered. By using the definition with margins free of HGIN, the complete resection rate increased to 87.6% in the current study. Even advanced endoscopic imaging may not overcome the problem of detection of small multifocal lesions close to the target area [22, 23]. Extended ESD with safety margins set at a greater distance from the detectable neoplasia margins may be required for a substantial improvement of the complete resection rate.

Post-ESD stricture is of great concern in the post-ESD management, and its incidence is reported to be 5% to 20.8% [16, 18, 20]. In the present study, we found no post-ESD stricture after ESD when a circumferential mucosal defect involved less than half the luminal circumference. The possibility of esophageal stricture increases if the mucosal defect is more than three-fourths of the esophageal circumference after ESD [24]. The stricture rate in the current study was 8.4% for all patients and 45.1% for patients in whom the lesion extended more than three-fourths of the luminal circumference. All strictures were successfully managed endoscopically. Steroid therapy to prevent strictures after ESD was not applied in our study, but recent studies have reported that steroids are useful for preventing strictures after ESD [25–27]. Apart from stricture, complications were rare and esophageal ESD was shown to be safe.

Residual and recurrence are important issues in curative treatment. The rates of residual and recurrence in the present study were consistent with the findings of previous studies on ESD in ESCNs [14, 20, 28]. However, the rates of residual and recurrence were relatively low when complete resection rate is taken into consideration. The possible reason for this result could be the coagulation damage of the specimen margin and the corresponding esophageal tissue margin during the dissection procedure [16]. Of note, all the residual and recurrence were successfully treated by APC, second ESD, or additional treatment.

This study has several limitations. First, this was a single-center, retrospective study. Therefore, the results may not be generalizable, and the retrospective nature of the review may have caused a potential bias in the analysis. However, the majority of the data was collected in a systematic way at the time the cases were performed, making the dataset relatively robust. Second, the mean follow-up period (30 months) was not sufficiently long for the evaluation of the long-term outcomes of ESD. Third, twenty-nine (9.8%) patients who underwent ESD did not have follow-up after the procedure; this may underestimate the rates of complications, residual, recurrence, and mortality in our cohort. Fourth, the fact that the ESD procedures were performed by five experts also poses a limitation to external validity.

In conclusion, our study showed that ESD is a successful and relatively safe treatment for intramucosal invasive ESCC and HGIN, fulfilling the criteria of lymph node negative tumors. This should encourage clinicians to select ESD performed by experienced operators as a potential or even preferred treatment option for lesions amenable to endoscopic treatment.

## Figures and Tables

**Table 1:** Patient and lesion characteristics.

	Combined	Intramucosal invasive ESCC	HGIN	*P* value
Number of patients	296	79	217	
Age (years)	61.4 (7.8)	61.4 (8.3)	61.4 (7.7)	0.947
Male/female	202/94	54/25	148/69	0.980
Hot-eating habit	141 (47.6%)	34 (43.0%)	107 (49.3%)	0.339
Number of lesions	307	79	228	
Location				
Cervical esophagus	0	0	0	
Upper thoracic esophagus	22 (7.2%)	6 (7.6%)	16 (7.0%)	
Middle thoracic esophagus	181 (59.0%)	46 (58.2%)	135 (59.2%)	
Lower thoracic esophagus	100 (32.6%)	27 (34.2%)	73 (32.0%)	
Abdominal esophagus	4 (1.3%)	0	4 (1.8%)	
Resected specimen diameter (cm)	3.0 (2.5–4.0)	3.8 (1.5)	3.0 (2.5–4.0)	0.008
Circumference of the lumen				
<1/2	223 (72.6%)	48 (60.8%)	175 (76.8%)	0.006
<3/4	33 (10.7%)	10 (12.7%)	23 (10.1%)	0.525
≥3/4	51 (16.6%)	21 (26.6%)	30 (13.2%)	0.006

ESCC: esophageal squamous cell carcinoma; HGIN: high-grade intraepithelial neoplasia.

**Table 2:** Endoscopic treatment/patients' clinical course after ESD.

	Combined	Intramucosal invasive ESCC	HGIN	*P* value
Number of lesions	307	79	228	
En bloc resection	287 (93.5%)	70 (88.6%)	217 (95.2%)	0.041
Complete resection	240 (78.2%)	58 (73.4%)	182 (79.8%)	0.235
Resection time (minutes)	60 (40–90)	60 (43–110)	60 (40–90)	0.487
Number of patients	296	79	217	
Length of hospitalization (days)	6 (5–7)	6 (5–8)	6 (5–7)	0.351
Follow-up period (months)	30 (13.3)	31 (12.3)	29 (13.8)	0.325
Complications				
Perforation	3 (1.0%)	1	3	
Bleeding	2 (0.7%)	0	2	
Stricture	25 (8.4%)	9	16	
Residual	27 (9.1%)	9 (11.4%)	18 (8.3%)	0.413
Recurrence	18 (6.1%)	6 (7.6%)	12 (5.5%)	0.702
Local recurrence	12	5	7	
Synchronous recurrence	4	1	3	
Metachronous recurrence	2	0	2	
Mortality	13	4	9	
Cancer-related death	0	0	0	

ESD: endoscopic submucosal dissection; ESCC: esophageal squamous cell carcinoma; HGIN: high-grade intraepithelial neoplasia.
